# Near-Single-Cell Proteomics Profiling of the Proximal Tubular and Glomerulus of the Normal Human Kidney

**DOI:** 10.3389/fmed.2020.00499

**Published:** 2020-09-17

**Authors:** Tara K. Sigdel, Paul D. Piehowski, Sudeshna Roy, Juliane Liberto, Joshua R. Hansen, Adam C. Swensen, Rui Zhao, Ying Zhu, Priyanka Rashmi, Andrew Schroeder, Izabella Damm, Swastika Sur, Jinghui Luo, Yingbao Yang, Wei-Jun Qian, Minnie M. Sarwal

**Affiliations:** ^1^Division of MultiOrgan Transplantation, Department of Surgery, University of California, San Francisco, San Francisco, CA, United States; ^2^Pacific Northwest National Laboratory, Biological Sciences Division, Richland, WA, United States; ^3^Environmental Molecular Sciences Laboratory, Pacific Northwest National Laboratory, Richland, WA, United States; ^4^Department of Pathology, University of Michigan, Ann Arbor, MI, United States

**Keywords:** glomerulus, mass spectrometry, single cell analysis, proteomics, kidney

## Abstract

Molecular assessments at the single cell level can accelerate biological research by providing detailed assessments of cellular organization and tissue heterogeneity in both disease and health. The human kidney has complex multi-cellular states with varying functionality, much of which can now be completely harnessed with recent technological advances in tissue proteomics at a near single-cell level. We discuss the foundational steps in the first application of this mass spectrometry (MS) based proteomics method for analysis of sub-sections of the normal human kidney, as part of the Kidney Precision Medicine Project (KPMP). Using ~30–40 laser captured micro-dissected kidney cells, we identified more than 2,500 human proteins, with specificity to the proximal tubular (PT; *n* = 25 proteins) and glomerular (Glom; *n* = 67 proteins) regions of the kidney and their unique metabolic functions. This pilot study provides the roadmap for application of our near-single-cell proteomics workflow for analysis of other renal micro-compartments, on a larger scale, to unravel perturbations of renal sub-cellular function in the normal kidney as well as different etiologies of acute and chronic kidney disease.

## Introduction

Recent advances in molecular profiling, specifically transcriptional analysis at a single cell level, can uncover rare cell populations within heterogeneous clinical tissues, which is contributing to our understanding of kidney biology, and its molecular processes (1–7). There is an unmet need to develop methods that can provide comprehensive biological information at the RNA and DNA levels and also allow for single-cell proteomic tissue analysis.

Proteomic technologies, unlike genomics, can provide functional information on cellular states and regulatory networks (2). A technological challenge for mass spectroscopy (MS) based proteomics is the limitation of starting material. Unlike transcriptomics, proteomics does not allow for molecular amplification. This factoid has resulted in substantive efforts to enhance the analytical sensitivity of MS-based proteomics, inclusive of technology miniaturization and higher efficiencies at the electrospray ionization source (8, 9), such that the analytical sensitivity is now sufficient to detect proteins in single mammalian cells. Despite having high analytical sensitivity, proteomic applications to small sample volumes has introduced additional challenges, such as non-specific adsorption of proteins and peptides to the surfaces of reaction tubes, inefficient digestion kinetics, the need for cleanup and challenges with delivery. Our group has recently reported on a near-single-cell proteomics (nscProteomics) method in HeLa cells, which provides a highly innovative and sensitive technology for proteome measurements of samples with sub-nanogram amounts of protein from a small number of cells (10). This method requires highly customized sample processing equipment, and is difficult to transfer out to other research labs in its current state of development. Processing human kidney tissues through this pipeline has required close attention to protocol development in order to optimize tissue preservation and cell capture.

In this study, we have focused on providing a roadmap for successful measurement of proteomic interrogation of various sub-compartments of kidney, at the near-single-cell level, with the following deliverables: (i) Assessment of optimal kidney tissue collection and storage for nscProteomics; (ii) Identification of the appropriate reference standard for nscProteomics; (iii) Optimization of the kidney tissue thickness for laser capture microdissection (LCM); (iv) Optimization of LCM parameters; (v) Description of the nscProteomics method using an LC-MS based, fully automated microPOTS method (μPOTS; Processing in One pot for Trace Samples) (11); (vi) Data analytics for nscProteomics.

**Figure 1:**
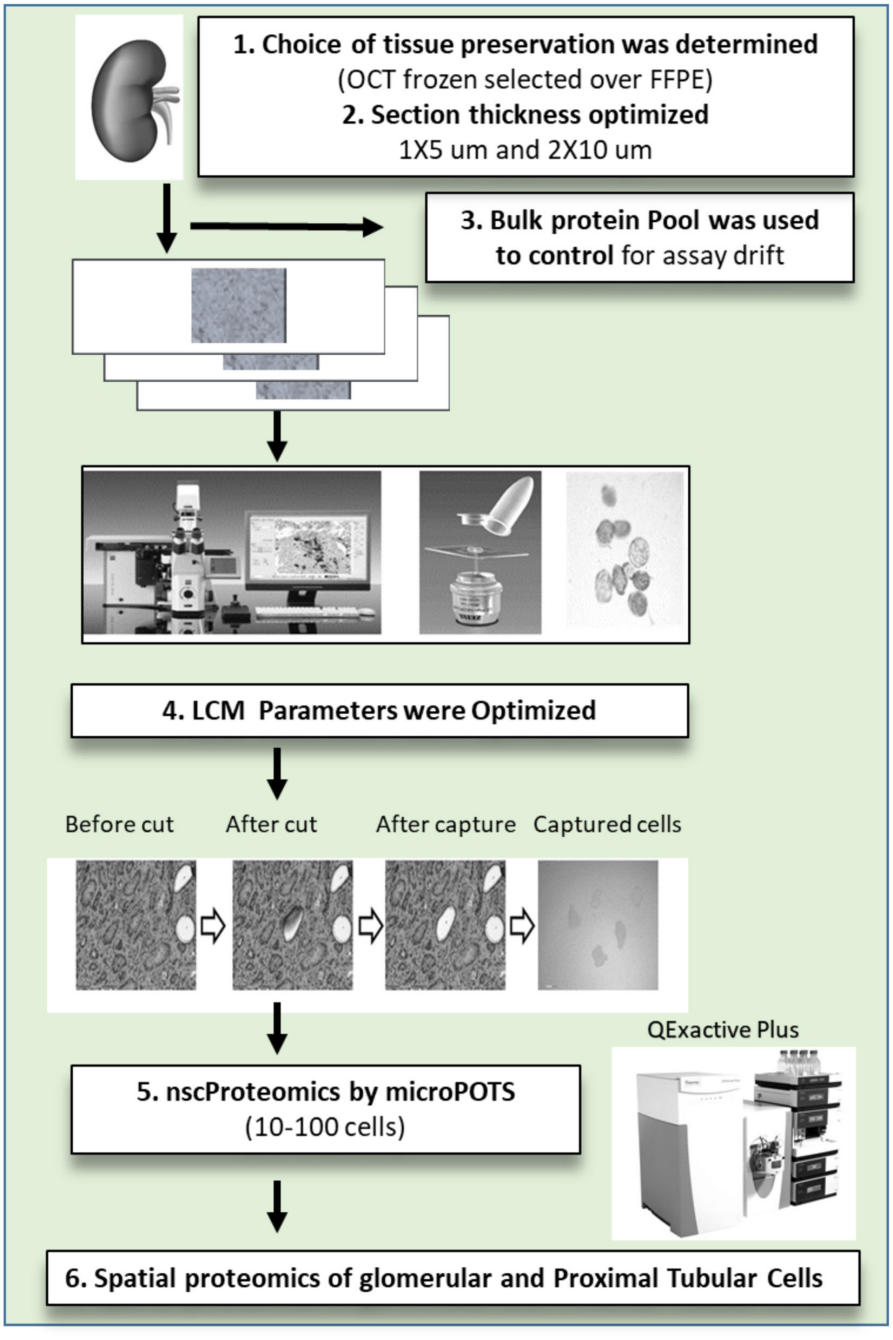
The near single cell proteomics (nscProteomics) workflow optimized for processing human kidney tissues. The workflow includes the identification of optimal kidney tissue collection and storage, the quality controls for laser capture microdissection establishment of nscProteomics method for kidney cells.

To achieve the aforementioned tasks, we have processed 11 unique human kidney biopsies and developed protocols and methodologies to collect, process, and interrogate proteomic expression of human kidney samples by μPOTS. When coupled with highly sensitive LC-MS, we exhibit a fully automated μPOTS method that enables reproducibility and provides quantitative proteomic measurements of ~3,000 proteins from 10 to 100 laser capture micro-dissected (LCM) kidney cells, a level of coverage only achieved previously for thousands of cells (12–17). This study will help for molecular characterization of tissue cellular heterogeneity and pathology in kidney biopsies from patients with different causes of acute and chronic kidney disease. This unique technology has the potential to unravel proteomic heterogeneity and unique protein markers in different kidney disease sub-types and sub-structures that will correlate to disease pathogenesis, prognosis and risk stratification. The study is summarized in Figure 1.

## Experimental Procedures

### Study Design

**Figure 2:**
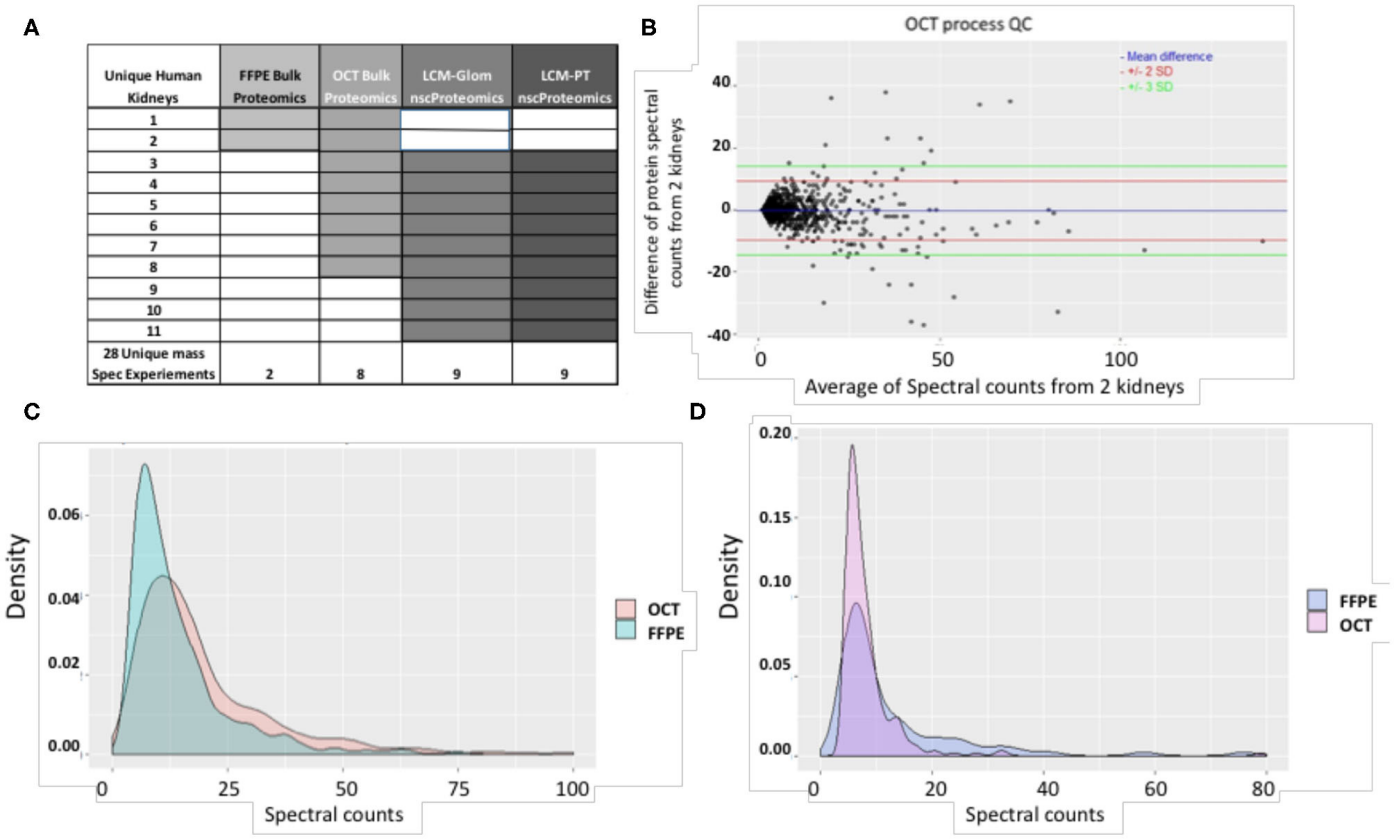
**(A)** A summary of study samples and assays. **(B)** The QC plot for reproducibility of the process using OCT tissue. Spectral count differences between proteins for 2 separate runs using OCT tissue was plotted against average protein spectral count for 1,257 proteins. In the plot, the blue line depicts mean difference; the red and green lines depict 2 SD and 3 SD limits respectively. 97.96% of proteins are within the calculated reproducibility limit of 13.52 indicating a high degree of reproducibility. It is also seen that the inter-run variability of the process involving OCT tissue is lower than that of the process involving FFPE tissue (calculated reproducibility limit: 24.43). **(C)** Comparison of spectral count distributions of 374 common proteins in OCT and FFPE. Larger number of proteins from OCT tissue show high spectral counts while a higher preponderance of low spectral counts is seen for proteins from FFPE tissue. **(D)** Comparison of spectral count distributions of 76 unique proteins in FFPE and 244 unique proteins in OCT. Higher preponderance of low spectral count proteins is seen in OCT tissue. This may indicate that OCT tissue technique is better for detection of low-abundant proteins in the current scenario. The correlation coefficient between proteins identified from 2 OCT frozen tissues was 0.96 (*P* < 0.001).

The schematic representation of the proteomic studies undertaken on 11 unique human normal kidneys is shown in Figure 2A. A total of 28 mass spectrometry (MS) runs were performed for bulk and laser capture micro-dissected (LCM) nscProteomics on paired glomerular (Glom) and proximal convoluted tubular (PT) sections. Tissues from the first two kidneys were preserved as FFPE and in optimal cutting temperature compound (OCT) medium. Kidney tissues issues from 9 more kidneys were only processed in OCT (Figure 2A).

### Kidney Tissue Sample Acquisition

Human kidney tissues were collected from total of 11 de-identified partial nephrectomies, obtained from the University of California San Francisco (UCSF) and University of Michigan (UM), with approved IRB, as part of a pilot technology feasibility study in the NIH Kidney Precision Medicine Project (KPMP). Only the healthy area of the kidney as determined by a trained pathologist was used for this purpose. Samples were anonymized with the only caveat being that the tissues were collected from adults. To assess the optimal sample collection protocol for the technology, initially ~100 mg of tissue from 2 kidneys was divided equally and either prepared as formalin fixed paraffin embedded (FFPE) tissue sections or was frozen in the Tissue-Plus™ O.C.T. Compound (Fisher Scientific). One 5 μm and three 10 μm thick consecutive sections were cut from each OCT block with a cryo-microtome and mounted on 1.0 polyethylene terephthalate (PET) membrane slides (Zeiss) for glomerular and proximal tubule dissection. The 5 μm thick section was H&E stained for visualization of major histological structures. The remaining 10 μm thick sections were left unstained. All slides were stored at −80°C until dissection.

### Assessment of Impact of Tissue Shipment and Processing on Proteomic Output

To assess the impact of delays on the proteomic analysis of OCT frozen tissue, kidney tissue samples were collected at one center, shipped to a second site >4 h travel away, and processed at the second center, and vice versa. Bulk proteomics was performed on two 2 unique kidneys (kidney #3, #4), procured at University of Michigan and shipped to 2 different locations (Ohio State University and UCSF) for MS analysis, using the same protocol and MS parameters at both sites. Results of the MS runs were then compared between the 2 sites.

### Protein Extraction and Precipitation

This was conducted on both the OCT and the FFPE tissue prep methods for selection of the optimal method for tissue proteomics. OCT tissue was sectioned into 10 μm thick sections using a cryomicrotome. To select the minimal amount of tissue needed for protein extraction for MS, three curls (a section that is put in an Eppendorf tube instead of spread on the slide), and 5 curls of frozen tissue were cut, thawed, and transferred to microcentrifuge tubes containing a 5 mm stainless steel bead (Qiagen), exposed to Mammalian Cell Lysis Buffer (including Benzonase^®^ Nuclease and Protease Inhibitor Solution; Qiagen), and homogenized in a TissueLyser LT (Qiagen) at 50 r/s for 3 min. Protein concentration was quantified (Bradford assay; Thermo Scientific) and stored at −20°C until use. Tissue stored for <2 months in FFPE was sectioned into ten 10 μm thick sections using a microtome. Three and five curls of tissue were cut, deparaffinized in xylene, centrifuged, and incubated in Extraction Buffer EXB1 (Qiagen) supplemented with β-Mercaptoethanol. Samples were incubated on ice, followed by incubation at 80°C for 2 h. Protein was quantified (Bradford assay; ThermoScientific) and stored at −20°C until further use. Protein was precipitated by the addition of cold acetone, followed by centrifugation to pellet and dry the precipitated protein. The pellet was re-suspended in 6 M Urea/100 mM Tris-HCl pH 7.8, and protein concentration was quantified (Bradford assay; ThermoScientific) and stored at −20°C until trypsin digestion.

### Trypsin Digestion

Approximately 150 μg of total protein (~15% of the available input) was exposed to reducing agents (200 mM DTT and 100 mM Tris-HCl) and alkylating reagents (200 mM iodoacetamide and 100 mM Tris-HCl) and then diluted with RNAse/DNAse free water to reduce Urea concentration to 0.6 M. Four μg of porcine Trypsin (Sigma) was added and the sample digested at 37°C. Protein concentration was quantified (Bradford assay; ThermoScientific) and 10 μg of protein was used for MS.

### MS for Bulk Proteomics to Assess the Optimal Kidney Tissue Processing Method

The tryptic peptide mixture was acidified with formic acid, cleaned with C18 Monospin columns and analyzed on the Orbitrap Q Exactive HF-X (Thermo Scientific). MS/MS was performed using Higher-Energy C-Trap Dissociation (HCD). Mass spectra were analyzed using Byonic v2.14.27 (Protein Metrics) allowing for 12 ppm mass tolerances. Variable post-translational modifications were allowed for, including oxidation, methylation, carbamylation, and phosphorylation. Proteins were limited to those scoring better than a 1% false discovery rate (FDR). Comparisons of protein abundance and MS data were made to select the optimal method for kidney tissue collection between FFPE and OCT.

### Laser Capture Microdissection

Unstained 5, 10, and 20 μM thick sections mounted on PET slides were placed on the stage of the Zeiss PALM MicroBeam Laser Microdissection system (Zeiss) to test optimal cutting thickness. Glomerular and proximal tubule regions were selected using the freehand tool and dissections were performed at 10X objective. Sections with ~4,580 μm^2^ area and 10 uM thickness was captured, which accounted for ~40 glomerular cells based on the previously published formula for estimation of glomerular cells in a healthy adult kidney (18). For the proximal tubular cells, we captured ~2,900 μm^2^ of proximal tubular cells, which accounted for ~36 cells. Cut sections were catapulted into a 200 μL opaque adhesive cap (Zeiss) (LPC Energy, 66; LPC Focus, 79) and stored at −80°C.

### Near-Single-Cell-Proteomics (nscProteomics)

To solubilize proteins collected in capture caps with LCM, a 10 μL droplet of 0.1% DDM 50 mM Tris pH 8 was added directly to the cap. Samples were incubated for 30 min at 37°C in an Eppendorf ThermoTop thermomixer to reduce evaporation, followed by centrifugation at 2,000 × g for 1 min to transfer the lysate to the vial. Proteins were reduced with 5 mM dithiothreitol and incubated at 37°C for 30 min. Alkylation was carried out at 10 mM iodoacetamide with a 45 min incubation in the dark at 25°C. Next, a 10 ng aliquot of Ls-C was added followed by 3 h incubation at 25°C. A 10 ng aliquot of trypsin was then added followed by overnight digestion at 25°C. The digested peptides were mixed with 15 μL aliquot of 18 MΩ water.

### LC-MS Platform for the Ultra-Small Samples

Peptide samples (25 μL) were separated using a 60 cm column, with a 50 μm inner diameter and an integrated emitter (New Objective). The columns were packed in-house with 1.7 μm diameter Waters BEH media. A 100-min gradient was produced using a Dionex Ultimate 3000 RSLC nanopump (Thermo Scientific). This system was coupled to a QExactive Plus mass spectrometer (Thermo Scientific). Mass spectra were collected from 300 to 1,800 m/z, using a top 12 data dependent acquisition method. MS1 spectra were collected with a mass resolution of 35K and MS2 with 17.5K. A 100 ms maximum IT was used to increase identifications from low abundance ions.

### Proteomic Data Extraction

All raw files were processed using MaxQuant (version 1.5.3.30) for feature detection, database searching and protein/peptide quantification (19) following settings described previously (10). Tandem mass spectra were searched in the UniProtKB/Swiss-Prot human database (downloaded on 29 Dec 2018 and containing 20,417 reviewed entries). The MS proteomics data for nscProteomics has been deposited to the ProteomeXchange Consortium via the PRIDE (20) partner repository with the dataset identifier PXD015058 and 10.6019/PXD015058.

### Data Analysis

MS data was obtained from runs performed at PNNL (nscProteomics) and Stanford University (bulk proteomics). For bulk proteomics. The following statistical methods were used for selecting the optimal kidney tissue collection method, either frozen in OCT or processed as FFPE, as assessed by quantitative tissue bulk proteomics, (i) Protein yield/mg of equivalent input tissue was calculated; (ii) MS reproducibility was tested on MS output from FFPE and OCT sections from each of 2 kidneys, prepared by the same individual using the same protocol, analyzed on the same MS instrument using a set protocol, a few days apart (21). A reproducibility limit (22) was used to provide an approximate bound on measurement differences between successive runs of a single process as well as an estimate of precision for comparison between processes. The correlation coefficient, which provided the degree of agreement between successive runs (using the same tissue), was calculated; (iii) Protein abundance and peptide fragment size distribution was calculated to assess variations originated from protein degradation, crosslinking due to formalin etc. Other aspects of QC, included (iv) assessment of process drift over replicates and time by using a common pool kidney reference and (v) calculation of bias. Stringent quantitative analysis and identification of unique proteins utilized data only with proteins with spectral counts ≥5 (23). Fisher's exact test was used to assess the enrichment of common and unique proteins mapped between two normal human kidneys, (#1 and #2). Based on the above analysis (see results), OCT frozen tissue was selected as the collection method for all subsequent analyses.

The next step of the data analysis focus was to identify, quantify, and validate protein sets enriched in or specific to the ~10–100 cells obtained from each of two selected kidney sub-compartments—Glom and PT regions, obtained from the same kidney, by bulk and LCM of OCT frozen kidney tissue (*n* = 9; kidneys #3–11) and run by nscProteomics.

A 2-step semi-conservative filtering approach was adopted to deal with issues of missing/incomplete data, and the G-test (24) was used to identify if the data was Missing At Random (MAR) or Missing Not At Random (MNAR). Additional data reliability assessment was done by stringently selecting proteins observed in ≥50% of all samples. *T*-test was used to identify for proteins significantly enrichment in glom or PT and multiple testing correction (Benjamini-Hochberg FDR) with significance threshold of 0.1 was applied. For purposes of the analysis, the relative intensity MS data (log 2 transformed) obtained from Glom, PT and bulk fractions from a single kidney were considered to be paired. We identified proteins enriched in Glom (with low levels in PT/ bulk), and unique to Glom (absent in PT) as well as proteins enriched in PT (with low levels in Glom/ bulk) and unique to PT (absent in Glom). Enrichment analysis was done only on data without missing values in each group to increase confidence. The correlation between the abundance values of common proteins was used to assess the degree of agreement between enriched sub-compartment proteins between separate runs of the same experiment. We also performed cross-validation of significantly enriched proteins in each compartment by parsing these comparisons from unique sample sets between 2 different runs, Run 1 and Run 2. Biological validation for enriched sub-compartment (Glom and PT) sets of proteins was undertaken by querying if known sub-compartment enriched proteins could be localized back to their specific regions in public data from the Human Protein Atlas (https://www.proteinatlas.org/) (25).

### Cross-Omics Data Comparison/Integration

To assess concordance of protein expression at the RNA level, data generated from single-cell RNA-sequencing (scRNA Seq) performed on 10 native nephrectomies was utilize, 9 of which were overlapping with the kidneys used in nscProteomics. For this 10x Genomics Chromium platform with v3 chemistry was used using the method optimized in the Sarwal lab as a Tissue Interrogation Site (TIS) of KPMP Consortium (a manuscript detailing the method and results is under preparation). For this cross-omics analysis we used transcriptome data from 45,411 kidney cells resolved into nine major cell types: Podocytes, mesangial cells, glomerular endothelium, stroma/interstitium, immune cells, proximal tubule, thick ascending limb of loop of Henle, distal/connecting tubule, and collecting duct. Analysis of single-cell data was done using the Seurat R package version 2.3.4. More in-depth analysis of this data is in development (26).

## Results

### OCT Frozen Kidney Collection Is the Preferred Method for Kidney Tissue Proteomics

For the same amount of input tissue, OCT frozen kidney yielded more protein than FFPE sections from the same kidney (OCT frozen: 1871 ± 92 vs. FFPE: 296 ± 10 ng protein; *p* < 0.0017) suggesting possible protein degradation in FFPE, despite the tissue being embedded in paraffin blocks for <2 months. Bulk proteomics data from the two human kidneys (#1, #2) demonstrated a significant overlap of identified proteins with 84% overlap between the 2 OCT kidneys (reproducibility limit = 13.52, 97.96% values within the reproducibility limit) and 83% overlap of the same kidney proteins between the 2 FFPE kidneys (reproducibility limit = 24.43, 98.5% values within the reproducibility limit) (Supplemental Table 1 and Figure 2B). Within each kidney, irrespective of the tissue storage method, ~70% of kidney structural (mostly extracellular matrix) proteins were conserved, suggesting that some proteins were more preferentially detected by each method. To evaluate what protein enrichment is seen between different collection methods, we found that OCT tissue proteomics yielded more proteins with high spectral counts ≥ 5 (odds ratio = 1.460601; 95% confidence interval = 1.238969, 1.722593; *p* = 4.472e-06), which may reflect better preservation of kidney tissue in OCT (Figure 2C). Proteomic analysis of OCT tissue also sampled more unique proteins as compared to FFPE tissue (odds ratio = 3.207089; 95% confidence interval = 2.371221, 4.370600; *p* = 4.985e-16) (Figure 2D). Additionally, many of these unique proteins were found to be of low-abundance and are more likely to be missed, even with very short preservation times of kidney tissue in FFPE. Irrespective of the tissue preservation method, we noted that there was strong overlap in common biological pathways. The pathways included small molecule metabolic processes (*p* = 2.59E-121 for OCT and *p* = 2.15E-109 for FFPE), carboxylic acid metabolic process (*p* = 3.62E-80 for OCT and *p* = 1.93 E-76 for FFPE), organic acid metabolic processes (*p* = 1.26E-76 for OCT and *p* = 3.62E-80 for FFPE) and drug metabolism processes (*p* = 7.90E-83 for OCT and *p* = 4.01E-75 for FFPE). This data highlights how the proteomics of kidney tissue is highly reproducible, irrespective of tissue preservation method for the two methods tested.

### Kidney Tissue Proteomics Is Highly Reproducible on Transported OCT Frozen Kidney Tissue

We analyzed data generated at the two sites (UCSF and Ohio State University) that followed the same protocol for performing bulk proteomics, as described in the methods section. Remarkably, ~70% proteins were commonly identified in the same kidney processed at two different sites with spectral count ≥ 5 (*r* = 0.95, *P* < 0.0001) (Raw data provided in Supplemental Table 2). More importantly, the two kidneys shipped from a central site produced highly correlated data (*r* = 0.89, *P* = 0.0001) (Supplemental Table 2), supporting that the tissue processing analytical protocols in place were quite robust.

### Optimization of Capturing of Kidney Cells Using Laser Capture Microdissection (LCM)

Varying thicknesses of OCT sections mounted on PET slides from the same kidney (*n* = 2) were tested at 5, 10, and 20 μm thickness, in triplicate, for LCM repeatability. The 10 μm section was found to be the most optimal on serial LCM testing, using the Zeiss PALM MicroBeam Laser Microdissection system (Cut Energy, 64; Cut Focus, 75). Cut cells were captured on adhesive caps. In addition to LCM process repeatability, we also found maximum capture efficiency of catapulted cells at 10 μm OCT section thickness.

### Proteomics of Sub-compartment Cells in Normal Human Kidney Identifies Enriched and Unique Glomerular and Proximal Tubular Proteins

**Figure 3:**
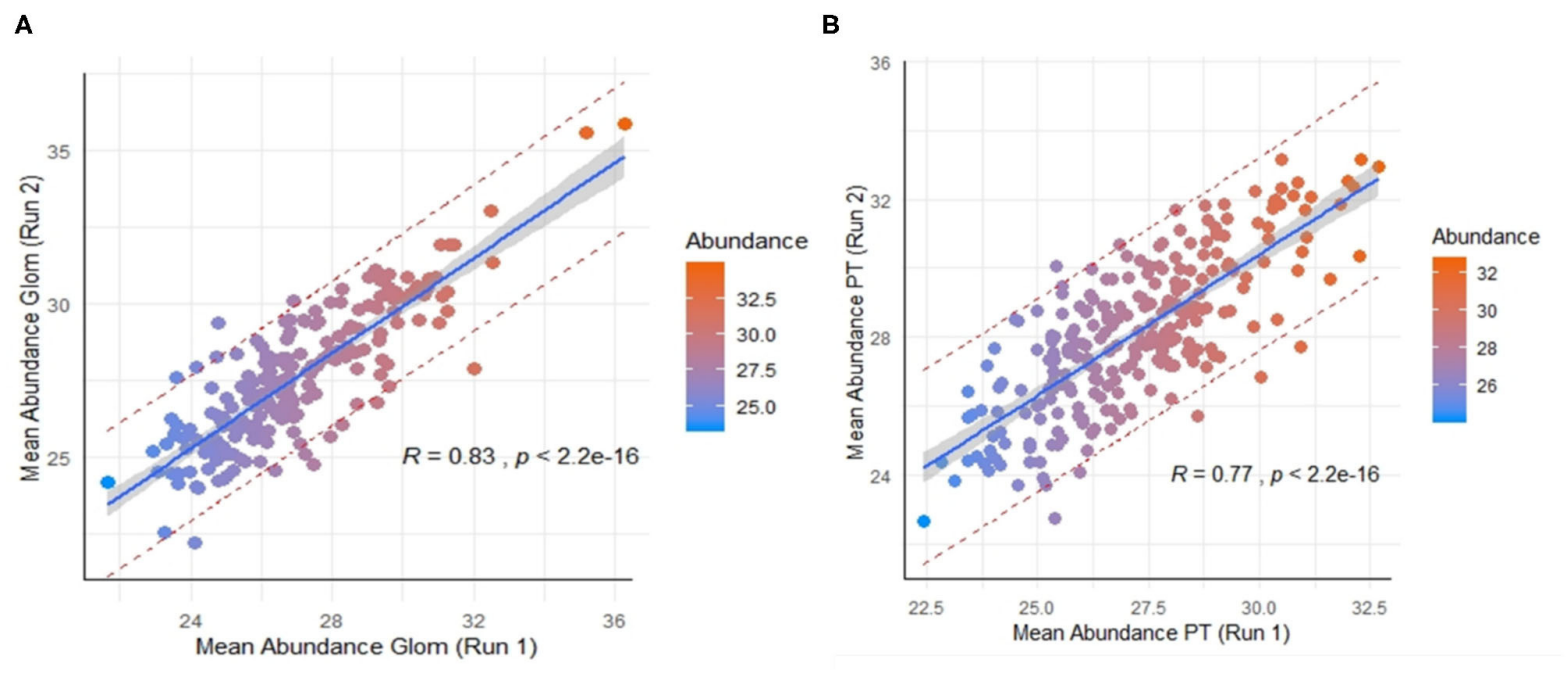
**(A)** Correlation of a set of 210 proteins significantly enriched in glomerulus between two different batches of independent kidney samples. Batch 1 (run 1) was from 2 kidneys and Batch 2 (run 2) was from 4 kidneys. Color scale was selected for visual depiction of abundance. A strong positive correlation of 0.83 between the mean abundances of the same set proteins from two different batches The red dashed lines give a measure of the spread by depicting the 95% prediction interval for mean abundance values of these proteins in future repeat experiments. **(B)** Correlation of 246 proteins significantly enriched in proximal tubules vs. glomerulus between the two batches. A positive correlation of 0.77 between the mean abundances of these same proteins between the two batches.

Across the 9 normal human kidneys processed by nscProteomics, with input of an average of 10–40 cells input each/Glom and PT fraction, we identified an average of ~2,560 proteins/sample. Interestingly, ~20% of the total proteins assessed by nscProteomics, from either sub-compartment overlapped across compartments/cells and bulk tissue, representing likely housekeeping or more structural kidney proteins that are not compartment specific. In a direct comparison of the two sub-compartments, 208 proteins were enriched (>2 fold) in Glom, and 67 were completely unique to Glom (absent in PT); and conversely, 247 proteins were enriched (>2 fold) in PT, and 25 were unique to PT (absent in Glom) (Supplemental Table 3). Despite the relatively small sample set, we also performed a cross-batch validation, treating each batch of runs as an independent set. Even with a small unique set of kidneys (2 in batch 1, 4 in batch 2), 210 Glom enriched proteins were highly correlated between the two independent sets, despite this data coming from different cohorts of normal kidneys (*r* = 0.83; Figure 3A) and analyzed at different times. 246 PT- enriched proteins could also be correlated between the two different cohorts of normal kidneys (*r* = 0.77; Figure 3B). The results highlight that nscProteomics is capable of identifying sub-compartment specific proteins conserved to different functional units of the kidney reproducibly.

To ascertain the specificity of the nscProtoemics approach for kidney sub-compartment analysis, we compared nscProteomics kidney sub-compartment Glom and PT data to bulk proteomics of the same set of kidney tissue. We found that ~25% (*n* = 656) of the proteins identified in both sub-compartments were not even detected by bulk tissue proteomics, as they were likely of very low abundanc e in the bulk sample; 118 Glom and 74 PT enriched proteins could not be detected in bulk kidney, highlighting the limitation of only doing bulk tissue proteomics. Only 9% of Glom-specific proteins, but a larger number of 48% PT-specific proteins, were identified by kidney bulk proteomics, reflecting the much higher abundance of PT cells over Glom cells in the normal kidney. In fact, some *known markers for glomerular cells* such as podocin (NPHS2), eva-1 homolog B (EVA1B), MARCKS like 1 (MARCKSL1), fibroblast growth factor 1 (FGF1), and claudin 5 (CLDN5) could not be identified by kidney bulk proteomics at standard run depths of ~2,000.

### Validation of Glom and PT Markers as Assessed by nscProteomics

**Figure 4:**
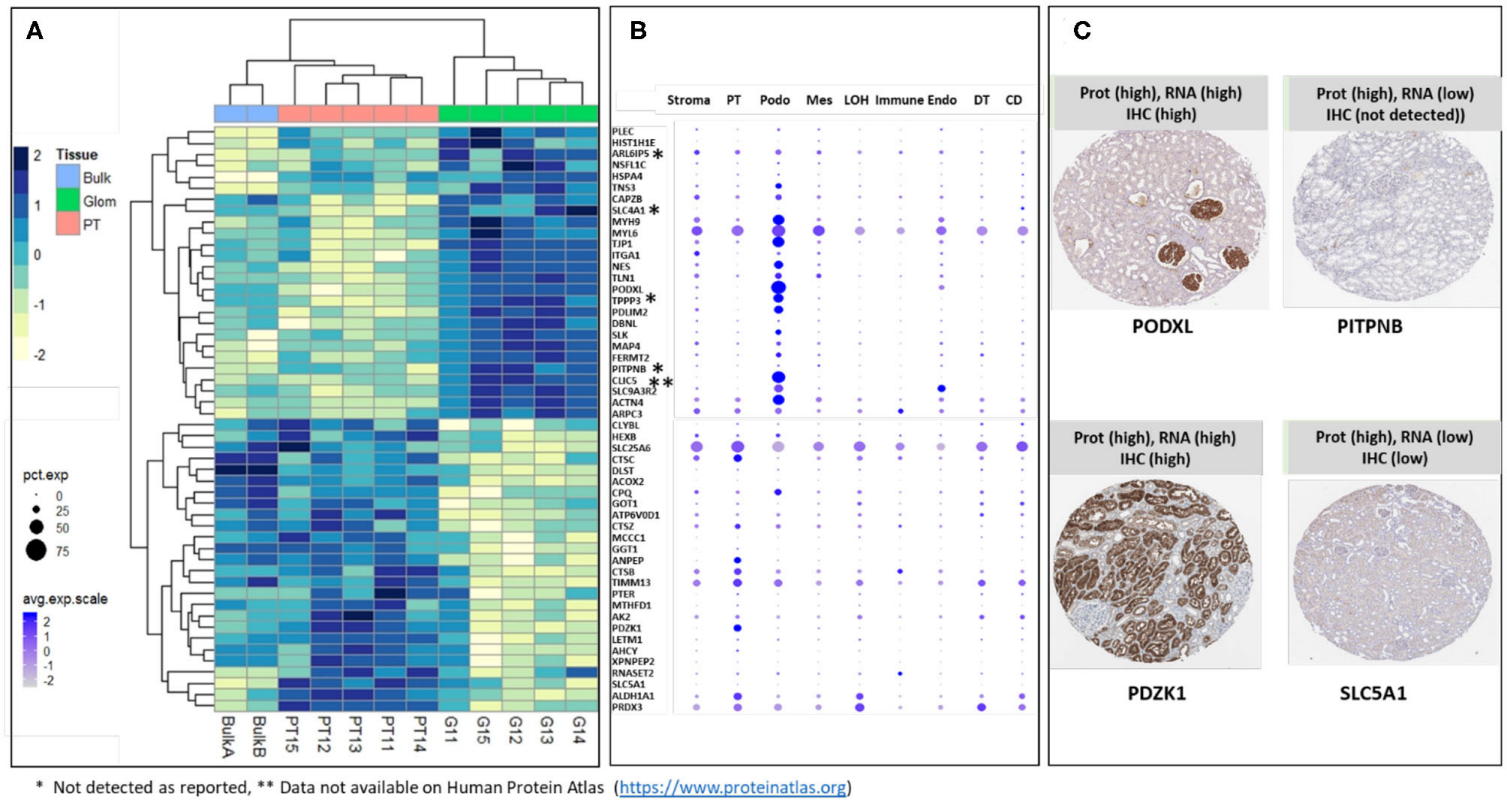
**(A)** Heatmap showing differential distribution of protein abundances in 5 normal kidneys (batch 2), of 26 proteins (out of 372) most significantly enriched in G (vs T) and 26 proteins (out of 411) most significantly enriched in T (vs G), with corresponding values in 2 Bulk samples shown for comparison. Protein abundances are measured as log 2 transformed relative intensities. Unsupervised clustering of significant proteins highlights sets of proteins related by Euclidean distance similarity measure within the larger differentiated enriched sets. It is noted that Bulk samples cluster together with PT samples; this is expected since proximal tubules are abundantly dispersed within the kidney. **(B)** Correlation of gene expression plot of scRNA-seq data from human kidney. Genes encoding Glom-specific and PT-specific proteins presented on the heatmap were selected. Increasing dot size corresponds to a larger percentage of cells in the cell population expressing the gene, while a darker color corresponds to a higher expression of the gene. PT, proximal tubular; Podo, podocytes; Mes, mesangial cells; LOH, Loop of Henle, Immune cells; Endo, endothelial cells; DT, distal tubules; CD, collecting duct. ^*^Sign next to the gene symbol indicates that the protein was not detected on glomeruli as reported by Human Protein Atlas (https://www.proteinatlas.org), ^**^Data not available on Human Protein Atlas (https://www.proteinatlas.org). **(C)** Representative proteins that demonstrate how nscProteomics not only identifies positive markers such as PODXL and PDZK1 but also identifies protein markers such as that are otherwise missed by transcriptomics or immunohistochemistry.

The majority of sub-compartment proteins were reported as detected in kidney by IHC in previously published data (Human Protein Atlas) (25). The proteins that were either not detected by IHC (labeled with ^*^) or data not available (labeled with ^**^) are indicated in Figures 4A,B. We took the top 26 glom enriched proteins and 26 PT specific proteins and interrogated single cell transcriptomic data (scRNA Seq data) for “cross-omics” integration and validation (Figure 4B). The result of integrative analysis of top Glom and PT markers by proteomics and transcriptomics demonstrated a strong correlation of some of the known Glom and PT markers proteins. Podocyte markers such as PODXL and CLIC5 and PT markers such as PDZK1 and ANPEP were highly enriched in both datasets. Apart from validating known markers, nscProteomics has also identified proteins that are identified in low abundance in either the transcriptomic datasets or IHC datasets. For example, PITPNB is a highly enriched glomerular marker by nscProteomics data; however, detection of this gene was low in transcriptomic data and was not detectableby IHC (25). A similar case was observed with SLC5A1, whose signal was enhanced in nscProteomics data but not in the transcriptomic or IHC datasets (Figure 4C).

### Biological Relevance of Glom and PT Enriched Proteins

We used the data generated through the developed protocol for nscProteomics to look at proteins that were significantly enriched in either the glomerular cells or the proximal tubular cells. The 208 Glom enriched proteins were enriched in vesicle-mediated transport (FDR 3.6E-15) and regulation of cellular component organization (FDR 9.75E-14) as their top biological processes and cytoskeletal protein binding (FDR 2.63E-19) and actin binding (FDR 2.40E-17) among the top ranked molecular function. Additionally, actin cytoskeleton (FDR 3.73 E-25) and focal adhesion (FDR 4.07E-20) were the top enriched cellular components. The 247 PT enriched proteins were enriched in small molecule metabolic processes (FDR 7.55E-50) and drug metabolic processes (FDR 5.00E-41) as top biological processes. The proteins were enriched for oxidoreductase activity (FDR 9.07E-34) and catalytic activity (FDR 1.05E-27) among the top ranked molecular functions.

### nscProteomics Identifies Unique Set of Glom and PT Proteins

Apart from enriched proteins in the Glom or the PT sections, 92 proteins were either unique to Glom (*n* = 67) or unique to PT (*n* = 25), as they were only identified in either the glomerular cells or the proximal tubular cells (Table 1). The proteins identified only in the glomerular cells were enriched in molecular functions such as actin binding (FDR 4.92E-06), cytoskeletal protein binding (FDR 3.47E-05), integrin binding (3.9E-03), etc. We used this list to interrogate the publicly available data that had previously reported their presence in the glomerulus. Among the 67 proteins identified only in Glom sections, 41 have been reported to be present in the kidney (25). Of these 41 proteins, a subset of 18 (43.9%) are in agreement with nscProteomics and 23 (56.1%) are reported to not be increased in Glom compared to tubules. Among the 25 proteins identified only in PT sections, 20 have been reported to be present in the kidney (25). These proteins are enriched in molecular functions such as organic anion transmembrane transporter activity (FDR 6.34E-05), carboxylic acid transmembrane transporter activity (FDR 1.4E-04), solute: sodium symporter activity (FDR 1.8 E-04), etc. Of the 25 PT specific proteins, a subset of 19 proteins (95.0%) are in agreement with nscProteomics and only 1 (5.0%) is reported to not be increased in tubules compared to Glom.

**Table 1:** Proteins enriched in glomerular and proximal tubular cells identified by nscProteomics.

**Glom or PT specific**	**Gene symbol**	**UniProtKB**	**UniProt ID**	**Protein name**	**Enrichment fold ± SD**
Glom	NDUFS5	O43920	NDSUFS5_HUMAN	NADH:ubiquinone oxidoreductase subunit S5	23.96 ± 1.02
Glom	SLC29A1	Q99808	S29A1_HUMAN	Solute carrier family 29 member 1 (Augustine blood group)	23.59 ± 0.46
Glom	GNG2	G3V415	G3V4N5_HUMAN	G protein subunit gamma 2	23.66 ± 0.39
Glom	MYO1D	O94832	MYO1D_HUMAN	Myosin ID	20.73 ± 1.60
Glom	GNG11	Q53Y01	Q53Y01_HUMAN	G protein subunit gamma 11	23.84 ± 0.25
Glom	FAM114A1	Q8IWE2	NXP20_HUMAN	Family with sequence similarity 114 member A1	23.16 ± 0.35
Glom	LGALS3BP	Q08380	LG3BP_HUMAN	Galectin 3 binding protein	21.78 ± 0.72
Glom	PYGL	P06737	PYGL_HUMAN	Glycogen phosphorylase L	22.13 ± 0.81
Glom	NEBL	O76041	NEBL_HUMAN	Nebulette	23.39 ± 1.78
Glom	RHOG	P84095	RHOG_HUMAN	ras homolog family member G	23.05 ± 0.78
Glom	RBM3	P98179	RBM3_HUMAN	RNA binding motif protein 3	23.39 ± 0.62
Glom	RCSD1	Q6JBY9	CPZIP_HUMAN	RCSD domain containing 1	22.40 ± 1.46
Glom	SYNPO2	Q9UMS6	SYNP2_HUMAN	Synaptopodin 2	25.31 ± 0.33
Glom	C1S	P09871	C1S_HUMAN	Complement C1s	21.58 ± 0.61
Glom	SLC2A1	P11166	GTR1_HUMAN	Solute carrier family 2 member 1	22.68 ± 0.84
Glom	C8B	P07358	CO8B_HUMAN	Complement C8 beta chain	23.29 ± 0.83
Glom	LSP1	P33241	LSP1_HUMAN	Lymphocyte specific protein 1	22.14 ± 0.64
Glom	TMEM150C	B9EJG8	T150C_HUMAN	transmembrane protein 150C	24.78 ± 0.46
Glom	LAMA5	O15230	LAMA5_HUMAN	Laminin subunit alpha 5	23.42 ± 1.86
Glom	ELANE	P08246	ELNE_HUMAN	Elastase, neutrophil expressed	22.21 ± 2.17
Glom	HLA-A	P04439	HLAA_HUMAN	Major histocompatibility complex, class I, A	22.78 ± 2.09
Glom	CACNA2D1	P54289	CA2D1_HUMAN	Calcium voltage-gated channel auxiliary subunit alpha2delta 1	22.69 ± 1.14
Glom	COL4A5	P29400	CO4A5_HUMAN	Collagen type IV alpha 5 chain	23.45 ± 0.65
Glom	CD109	Q6YHK3	CD109_HUMAN	CD109 molecule	21.81 ± 1.21
Glom	GMFG	O60234	GMFG_HUMAN	Glia maturation factor gamma	23.76 ± 0.95
Glom	SMCO3	A2RU48	SMCO3_HUMAN	Single-pass membrane protein with coiled-coil domains 3	23.30 ± 1.24
PT	DES	P17661	DESM_HUMAN	Desmin	23.42 ± 0.47
PT	MISP3	Q96FF7	MISP3_HUMAN	MISP family member 3	22.57 ± 0.37
PT	SLC5A8	Q8N695	SC5A8_HUMAN	Solute carrier family 5 member 8	23.83 ± 1.84
PT	SLC17A3	O00476	NPT4_HUMAN	Solute carrier family 17 member 3	23.52 ± 1.75
PT	UGT2A1	Q9Y4X1	UD2A1_HUUMAN	UDP glucuronosyltransferase family 2 member A1 complex locus	23.21 ± 1.35

## Discussion

With rapid advancement of single cell genomics and transcriptomic assays, there is still an unmet need to do parallel single cell studies on precious human samples in the field of proteomics (27). Protein profiling from single cell faces hindrance from the virtue that we do not have protein amplification capability the way we can amplify nucleotides. In this first application of near-single-cell proteomics on the human kidney, we demonstrate that robust label-free proteomic quantification is feasible for as few as 40 kidney cells, specific to different LCM sub-compartments. The developed protocols for near-single-cell kidney proteomics will be of benefit to other researchers working in the field of kidney diseases.

A recent study by Hoehne et al. demonstrated the use of Single-Pot Solid-Phase-enhanced Sample preparation (SP3) technology for proteomic analysis of renal segments (4). Using single microdissected glomeruli with ~200 cells the authors illustrated the heterogeneity of the proteomes from single glomeruli from mouse models and human patients. Another recent study by Song et al. (28) utilized FFPE tissue from renal transplant recipients to create a molecular diagnostic platform using bulk proteomics. In this manuscript, we have optimized and analyzed in great detail the process of tissue collection to MS to establish a standard protocol for proteomic analysis of small tissue samples. First, we compared the FFPE vs. OCT method of tissue storage to show that while there is a high correlation between the two datasets, OCT tissue yield higher protein content and more importantly the ability to identify low abundance proteins. Second, MS protocols for bulk proteomics have been optimized such that highly reproducible (*r* = 0.95) results can be obtained at disparate sites on serially cut, adjacent kidney tissue sections. Third, were we describe an approach to couple laser capture microdissection to reduce the amount of input sample to 10–40 cells with a nanoPOTS technology consisting of a robotic platform to accurately handle pico-liter volumes. Our approach allows us to control the input number of cells thus enhancing the reproducibility of between different glom and tubule samples. Due to the simplicity of LCM and the accuracy of nanoPOTS technology, we believe that our study will allow the adoption of proteomics on precious clinical specimens in a reproducible manner.

Limitations of this study include the small sample size and the limitation of tissue which did not allow us to do tissue spatial validation by immunohistochemistry. To partially alleviate this limitation, we provide integrated data analysis with scRNA Seq data from the same tissues to confirm that proteins identified in kidney sub-compartments are also identified in the same originating tissue cell by transcription. An additional limitation is that we identify a restricted number of proteins by nscProteomics, likely due to small input material, which runs the risk of some proteins being not identified by MS as they could like below the limit of instrument detection. Improvement in instrument sensitivity and protein extraction from small sample input may help improve this current limitation.

We acknowledge that there are many variables that can contribute to the quality of the data. The storage time, FFPE fixatives (i.e., PFA vs. Glutaraldehyde), length of time in fixative, time in the freezer/on dry ice/in liquid nitrogen, approaches for protein solubilization are a few factors that could contribute in proteomics results. It is important to realize that even biology of two kidneys is not always the same and that is true even within the same kidneys as glomeruli of same kidney biopsies can be at functional different state. Histologically, kidney compartments are extremely heterogeneous even within the same disease condition. By capturing the same regions of the kidney from different input samples, nscProteomics allows for interrogation of heterogeneity within specific sub-cellular compartments, which is a strength of this technology.

We observed that some sub-compartment proteins identified by nscProtoemics are potentially novel glomerular and proximal tubular proteins. Innovations in integrative data analysis with single-cell RNA Seq of the same kidney samples, though preliminary, would provide confirmation that these proteins are indeed localized to their sampled/predicted sub-regions.

This study provides insights into the undiscovered complexity of molecular patterns in kidney sub-compartments in health and supports the application of this technology to kidney disease samples to understand the perturbation of proteins in different kidney sub-compartments that may be preferentially affected in different kidney disorders. Differential regulation of these proteins in different renal diseases could uncover clinical and prognostic heterogeneity in complex glomerular and tubular disease phenotypes and uncover new triggers of kidney damage and recovery. In addition, given the paucity of any renal protective drugs, uncovering these new pathways in specific regions of the kidney can provide important new targets for rational drug design for addressing specific injures to different sub-compartments in a variety of renal disorders.

Thus, in conclusion, we present a nscproteomics workflow for handling 10–40 kidney cells; improvement in this technology carry the promise to reduce input material in the future to single cell level. Future developments such as optimized processing volumes and further refinements to the LC-MS platform are underway.

## Data Availability Statement

The datasets generated for this study can be found in the PRIDE PXD015058 and 10.6019/PXD015058.

## Author Contributions

TS and MS conceptualized the study. TS, JLi, JH, ACS, RZ, YZ, PR, ID, SS, JLu, and YY helped in sample processing and experimental data generation, TS, PP, SR, AS, W-JQ, and MS contributed in data analysis and manuscript preparation. All authors contributed to the article and approved the submitted version.

## Conflict of Interest

The authors declare that the research was conducted in the absence of any commercial or financial relationships that could be construed as a potential conflict of interest.

## Abbreviations

nscProteomics: near-single-cell proteomics
KPMP: Kidney Precision Medicine Project
PT: proximal tubular
Glom: glomerular
LCM: laser capture microdissection
μPOTS: Microliter Processing in One pot for Trace Samples
OCT: Optimal cutting temperature
FFPE: formalin fixed paraffin embedded
UM: University of Michigan
OSU: Ohio State University
DDM: n-dodecyl β-D-maltoside
scRNA Seq: single cell RNA Sequencing
IHC: immunohistochemistry.
