# Corrigendum: Near-Single-Cell Proteomics Profiling of the Proximal Tubular and Glomerulus of the Normal Human Kidney

**DOI:** 10.3389/fmed.2020.625788

**Published:** 2021-01-12

**Authors:** Tara K. Sigdel, Paul D. Piehowski, Sudeshna Roy, Juliane Liberto, Joshua R. Hansen, Adam C. Swensen, Rui Zhao, Ying Zhu, Priyanka Rashmi, Andrew Schroeder, Izabella Damm, Swastika Sur, Jinghui Luo, Yingbao Yang, Wei-Jun Qian, Minnie M. Sarwal

**Affiliations:** ^1^Division of MultiOrgan Transplantation, Department of Surgery, University of California, San Francisco, San Francisco, CA, United States; ^2^Pacific Northwest National Laboratory, Biological Sciences Division, Richland, WA, United States; ^3^Environmental Molecular Sciences Laboratory, Pacific Northwest National Laboratory, Richland, WA, United States; ^4^Department of Pathology, University of Michigan, Ann Arbor, MI, United States

**Keywords:** glomerulus, mass spectrometry, single cell analysis, proteomics, kidney

The Kidney Precision Medicine Project (KPMP) Consortium was incorrectly included as an author in the published article, as indicated by the phrase “Minnie M. Sarwal^1*^ and the Kidney Precision Medicine Project (KPMP) Consortium.” The correct phrasing is “Minnie M. Sarwal^1*^ for the Kidney Precision Medicine Project (KPMP) Consortium.”

The authors apologize for this error and state that this does not change the scientific conclusions of the article in any way. The original article has been updated.

